# Ribosome Inactivating Proteins: From Plant Defense to Treatments against Human Misuse or Diseases

**DOI:** 10.3390/toxins10040160

**Published:** 2018-04-18

**Authors:** Julien Barbier, Daniel Gillet

**Affiliations:** Service d’Ingénierie Moléculaire des Protéines (SIMOPRO), CEA, Université Paris-Saclay, LabEx LERMIT, 91191 Gif-sur-Yvette, France; julien.barbier@cea.fr

Ribosome inactivating proteins (RIPs) form a vast family of hundreds of toxins from plants, fungi, algae, and bacteria. RIP activities have also been detected in animal tissues. They exert an N-glycosydase catalytic activity that is targeted to a single adenine of a ribosomal RNA, thereby blocking protein synthesis and leading intoxicated cells to apoptosis. In many cases, they have additional depurinating activities that act against other nucleic acids, such as viral RNA and DNA, or genomic DNA. Although their role remains only partially understood, their functions may be related to plant defense against predators and viruses, plant senescence, or bacterial pathogenesis.

In this Special Issue, a review by Fabbrini and colleagues [1] addresses our current knowledge about the function and mechanisms of action of plant type I and type II RIPs. In particular, they emphasize the diversity found in their mechanisms of action, although they share sequence and structural identities in their catalytic chain. In another review, De Zaeytijd and Van Damme focus on the heterogeneity of cereal RIPs from an evolutionary perspective, their differences from non-cereal RIPs, and their variety of roles in addition to defense against pathogens and insects [2].

Most RIPs are no threat to human or animal health. However, several bacterial RIPs are major virulence factors involved in severe epidemic diseases such as dysentery or the hemolytic uremic syndrome that may occur in patients suffering from Shiga toxin-producing entero-hemorrhagic *Escherichia coli* infection. A few RIPs synthesized in plant seeds such as ricin toxin, abrin, or sarcin have been or may be involved in accidental or criminal poisonings, political intimidation, or bio-suicides. In this Special Issue, four contributions address the most recent advances in understanding the three major steps of the intoxication process of cells by Shiga toxins, ricin, and/or sarcin: receptor-binding and triggering of endocytosis, the components of the intracellular trafficking machinery involved in intoxication and binding, and depurination of the ribosome. Johannes describes how the pentavalent binding of Shiga toxins to Gb3 gangliosides in lipid rafts induces membrane structural changes and stress leading to the internalization of the toxin-receptor complexes [3]. Becker and colleagues set up an elegant screening method in yeast, enabling them to not only to confirm the importance of the GARP complex and other protein partners in ricin A chain intracellular trafficking, but also to identify seven new proteins involved along the pathway [4]. This method can now be applied to identify trafficking components used by other RIPs. Li and Tumer analyze the differences in ribosome binding and catalytic activities of the non-activated and activated Shiga and ricin holotoxins, showing opposite behaviors for these two toxins [5]. Finally, Shi et al. describe the structure of the complex of ricin A chain with the C-terminal peptide of the ribosome stalk protein P2 [6]. They discuss the differences in this interaction with that of other RIPs with the same ribosomal target.

The pathophysiology of intoxication by the most dangerous RIPs, such as Shiga toxins and ricin toxin, seem much more complicated than a sole link to circulation in the bloodstream and cell death, and is still far from being understood. Diana Karpman and her group review their recent work that brings important progress in the understanding of the mechanisms underlying the hemolytic uremic syndrome provoked by Shiga toxins [7]. They show that Shiga toxins are internalized by red blood cells and then released in microvesicles. It is these toxin-containing microvesicles that participate in the prothrombotic lesions, hemolysis, and transfer of the toxin from the circulation into the kidney, that are characteristic of this deadly syndrome. Furthermore, a research article from the groups of Lee and Park describes for the first time the apoptotic processes induced by Shiga toxins in human retinal pigment epithelial cells, suggesting the mechanisms leading to blindness in severe cases of hemolytic and uremic syndrome [8]. Gal et al. extend their characterization of the crucial role of inflammation in ricin toxin pathogenesis by showing that total body irradiation of mice decreases inflammation markers and extends time to death [9]. Second after ricin toxin, the plant RIP abrin is considered an increasing risk of malevolent and suicidal use. Thus, there is a need for detection and decontamination tools. Tam et al. set up a two-monoclonal antibody-based ELISA that can detect as low as 1 ng/mL of abrin and that shows no false positive detection of other plant RIPs [10]. The same team showed that while various pH treatments of the toxin did not affect its activity, heating above 74 °C completely inactivated its capacity to kill cells and mice. However, they showed that this treatment affects the lectin part of the toxin rather than its catalytic chain [11]. Interestingly, this article sets a correspondence between cytotoxicity testing and the mouse bioassay, which should help reduce the use of the mouse model for the evaluation of abrin and other RIPs. 

Due to sporadic but recurrent cases of biosuicide and biothreats with ricin toxin, there is an urgent need for a treatment of human intoxication. The group of Kronman gives us a thorough review of existing data on potential countermeasures and treatment strategies, although none are approved for medical use [12]. While antibodies represent the most realistic approach in the case of early post-intoxication intervention, the review stresses the importance of not only eliminating the toxin but also downregulating the explosive inflammatory response triggered by the toxin, as additionally described in a research article by the same group [9].

Interestingly, Whitfield and colleagues describe in detail an F(ab’)_2_ polyclonal ovine antitoxin and its performance in a mouse model of ricin inhalation that is intended to be pharmaceutically qualified for human use [13]. Protection is mediated both by reducing the amount of circulating toxin and blocking its intracellular trafficking to the Golgi apparatus. 

Many studies in the past showed that a fraction only of the antibodies generated in the course of an immune response against ricin toxin was neutralizing. Here, the group of Nicholas Mantis identifies the presence of a supercluster of neutralizing epitopes at the interface between the A and B chains of the toxin by analyzing a series of V_H_H camelid antibody fragments from a phage library generated against ricin toxin [14]. Interestingly, these antibodies do not interfere with the binding of the toxin to the galactose and N-acetyl-galactosamine residues of cell surface glycosylation. This is a step forward in understanding the basis for antibody-mediated protection against this toxin.

Hall et al. review the attempts to develop antibodies or other antitoxin strategies to treat the hemolytic and uremic syndrome caused by Shiga toxins, none of which have reached approval [15]. They suggest that the rarity of this disease is a major limit to achieving the necessary clinical trials. Then they advocate the development of drugs targeting the unfolded protein response and the ribotoxic stress response triggered by Shiga toxins as these pathways are involved in many other conditions, which may decrease the barriers to commercial development. 

The final aspect of research on RIPs covered by this Special Issue concerns their use in the engineering of immunotoxins to target cancer or cells infected by Human immunodeficiency virus (HIV) by conjugation of antibodies or other targeting moieties. Two reviews by Fabbrini et al. and Rust et al. discuss the difficulties that have been encountered in the development of several generations of immunotoxins, none of which have been approved after clinical trials [1,16]. They also present the future trends of immunotoxin development. Two examples of the complexities of such development are given in the articles of Polito et al. and Au et al. The former analyzes the difference in the mechanism of killing of two closely related saporin-containing immunotoxins targeting different markers on B-cell lymphomas, CD20 and CD22 [17]. The latter addresses the effect of PEGylation on the pharmacology, biological activity, and antibody induction of a TAT-maize RIP construction designed to target HIV-infected cells [18].

Overall, this Special Issue of *Toxins* presents the most recent data on all aspects of RIPs, including function, diversity, evolution, as well as mechanism, pathophysiology, medical countermeasures, and engineering into anticancer drugs.
